# Risk Factors for and Prevalence of Failure of Passive Transfer of Immunity in Calves on Dairy Farms in Türkiye

**DOI:** 10.1002/vms3.70919

**Published:** 2026-04-03

**Authors:** Nezaket Sağlam, Onur Topal, Piraye Biçer, Ender Uzabacı, Hıdır Gençoğlu, John F. Mee, Hasan Batmaz

**Affiliations:** ^1^ Department of Internal Medicine Faculty of Veterinary Medicine University of Bursa Uludağ Bursa Türkiye; ^2^ Laboratory and Veterinary Health Program Karacabey Vocational School, University of Bursa Uludağ Bursa Türkiye; ^3^ Bayfeed Consultancy İzmir Türkiye; ^4^ Department of Biometrics Faculty of Veterinary Medicine, University of Bursa Uludağ Bursa Türkiye; ^5^ Department of Animal Nutrition and Nutritional Diseases Faculty of Veterinary Medicine, University of Bursa Uludağ Bursa Türkiye; ^6^ Animal & Bioscience Research Department Teagasc Moorepark, Fermoy, County Cork Carlow Ireland

**Keywords:** calves, dairy cows, herd size, transfer of passive immunity

## Abstract

**Background:**

Failure of passive transfer of immunity (FPTI) is perhaps the most important factor in the health of neonatal calves. Given the variation between countries in FPTI, national data need to be documented to add to the international database for future meta‐analyses.

**Objectives:**

The aim of this study was to evaluate the prevalence of and risk factors for FPTI in dairy herds in Türkiye.

**Methods:**

Blood serum samples from 1026 calves in 76 dairy herds in 16 provinces of Türkiye were analysed using a digital Brix refractometer to assess FPTI, defined as a Brix value < 8.5%. Data on risk factors were collected during farm visits to sample calves. Herd size was categorised based on the number of lactating cows: Group I (≤ 20), Group II (21–100), Group III (101–200), Group IV (201–300), Group V (301–500), Group VI (501–1000) and Group VII (> 1000).

**Results:**

FPTI incidence was 22.4% overall. Significant factors associated with FPTI included small herd size (*p* = 0.002), lower dam parity (*p* = 0.005), breed of calves (*p* < 0.001), natural sucking of colostrum (*p* = 0.003) and birth in summer or winter (*p* = 0.005).

**Conclusion:**

The prevalence of FPTI in Turkish dairy herds was within international norms but higher than recently recommended consensus prevalence. This research shows that there were both modifiable and non‐modifiable significant risk factors for FPTI in Turkish dairy herds, specifically colostrum feeding method and season of birth and herd size, dam parity and calf breed.

## Graphical abstract

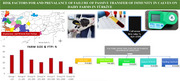

## 1 Introduction

One of the most critical issues facing the global dairy cattle industry is the improvement of calf health within the first few days of life. Calves are born agammaglobuliniaemic, necessitating the intake of colostrum, a rich source of nutrients, growth factors and immunoglobulins, particularly immunoglobulin G (IgG), in order to establish passive immunity (McGrath et al. 2016; Godden et al. 2019; Mee 2023). It is recommended that calves be administered colostrum as soon as possible following birth, prior to the process of gut closure, to prevent systemic infections. This is because the closure of the gut prevents the absorption of large molecules, including immunoglobulins, after this point in time. The process of calf gut closure typically occurs approximately 24 h after birth, although this timing can vary (Godden et al. 2019; Batmaz 2021).

Factors that influence the efficacy of the transfer of passive immunity (TPI) in calves include the quantity and quality of colostrum, the timing of its administration, dry period length, the age and parity of the dam and the presence of the dam (Weaver et al. 2000; Godden 2008; Hue et al. 2021).

When evaluating feeding methods, colostrum intake can be accurately measured in bottle and oesophageal tube‐fed calves; however, the volume consumed by suckling calves remains uncertain. The higher failure of passive transfer of immunity (FPTI) rate observed in suckling calves may be attributed to the unknown quantity and quality of colostrum ingested (Godden et al. 2019). Although oesophageal tube feeding offers advantages, it is a technically challenging procedure and poses risks such as aspiration, which may lead to complications (Phipps et al. 2018).

Control of colostrum leakage pre‐ and intra‐partum, early colostrum milking and ensuring that calves receive sufficient colostrum in the first hours of their lives are methods that can reduce the rate of FPTI (Reschke et al. 2017). In relation to the quantity of colostrum that should be consumed by the calf, while some suggested that 8.5% of the calf's body weight should be given within the first 2 h after birth (Conneely et al. 2014), Chigerwe et al. (2009) suggested that at least 3 L of it should be given within 4 h after birth and that calves unable to drink should be given by oesophageal intubation. There are divergent recommendations for the recommended dosage of colostrum to be administered to a newborn calf (Conneely et al. 2014).

Environmental factors such as calving season and temperature–humidity index have been shown to affect colostrum quality, so these factors may influence FPTI (Zentrich et al. 2019; Gulliksen et al. 2008).

While the factors affecting FPTI at the herd level were investigated in previous years, herd size was also investigated within these factors. The FPTI ratios were 51.95%, 17.2%, 22.1% when listed according to the number of cows < 100, 100–500 and > 500, respectively (Beam et al. 2009).

There are studies showing that gender affects the FPTI rate in calves and that this rate is lower in female calves than in males (Bragg et al. 2020; Mellado et al. 2024). Given the same amount of colostrum, male calves are more likely to have FPTI because their birth weight is higher than that of females (Mellado et al. 2024).

In the study conducted by Reschke et al. (2017), the FPTI rate in calves was found to decrease significantly as the time of first colostrum intake shortened (< 2 h; 2–6 h; > 6 h). In another study, daily IgG concentrations were measured in calves and a high correlation was found between serum IgG concentrations at 24 h and those on Days 2 and 3, a moderate correlation between Days 4 and 9 and a low correlation on Day 10. These results concluded that calves can be reliably tested for FPTI rates up to 9 days of age (Wilm et al. 2018).

The examination of calves for FPTI represents a crucial phase in the evaluation of the efficacy of colostrum management programs and the investigation of persistent calf health issues (Morrill et al. 2012; McGuirk 2008). FPTI is defined as the inadequate absorption of colostral immunoglobulin (Ig) by the calf during their immediate postnatal period. A concentration of IgG below 10 g/L is indicative of FPTI (Sutter et al. 2020; Lima et al. 2024; Silva et al. 2025). The assessment of FPTI is typically conducted during the initial week of life (Godden 2008; Sutter et al. 2020).

Several methods have been developed to measure IgG concentration in dairy calves either directly or indirectly (Elsohaby et al. 2019; de Souza et al. 2021). The direct methods include the use of radial immunodiffusion (RID) assays, enzyme‐linked immunosorbent assays (ELISAs) and infrared spectroscopy (de Souza et al. 2021; Alley et al. 2012). The indirect methods include the use of refractometry (e.g., Brix, total protein), gamma‐glutamyl transferase test (GGT), the zinc sulfate (ZnSO) turbidity test, glutaraldehyde coagulation test and sodium sulfate turbidity test (Batmaz 1992; Weaver et al. 2000; Topal et al. 2018; Godden et al. 2019; Constantin et al. 2023).

The Brix refractometer, which is fast, reliable, inexpensive and easy to use, is the most widely used tool in the field to monitor TPI (Deelen et al. 2014; Godden et al. 2019). In previous studies conducted to evaluate FPTI in dairy calves, the Brix cut‐off value (and correlations with RID) was determined as 8.4% (Se [88.9%] Sp [88.9%]; *r* = 0.93) by Deelen et al. (2014); 8.5% (Se [100%] Sp [89.2%]; *r* = 0.79) by Hernandez et al. (2016); 8.4% (Se [96.3%] Sp [88.8%]; *r* = 0.89) by Akköse et al. (2022b) and 10% (*r* = 0.74) by Thornhill et al. (2015) can be used as cut‐off values for the determination of FPTI in calves (Deelen et al. 2014; Thornhill et al. 2015; Hernandez et al. 2016; Akköse et al. 2022a).

A review of the literature reveals the absence of a large‐scale study that comprehensively addresses FPTI in calves in Türkiye in a manner that reflects the country. In this regard, the present study is one of the most comprehensive studies conducted nationwide. The aim of this study was to investigate the prevalence and risk factors affecting FPTI in dairy herds in Türkiye.

## 2 Material and Methods

This study was approved by the Local Ethical Committee for Animal Research of XXX University (2023‐13/04).

### 2.1 Animals and Herds

A total of 1026 dairy calves from 76 herds in 16 provinces in five regions of Türkiye were blood‐sampled once between March 2023 and February 2024. This was a convenience sample of herds selected to be of different sizes, typical of Turkish dairy herds. The provinces where the blood samples were collected and the farms, grouped according to their size and the number of sampled calves from these farms, are shown in Table 1. Herd sizes ranged from 2 to 3800 dairy cows.

**TABLE 1:** Distribution of calf serum samples by regions, provinces and herd size groups.

Regions	Provinces	Herds (*n*)	Calf samples (*n*)/group							
			GI	GII	GIII	GIV	GV	GVI	GVII	Calf samples total (*n*)
Marmara	Balıkesir	8	7	5	–	–	40	14	–	66
	Bursa	16	20	12	1	48	85	92	14	272
	Çanakkale	2	–	–	–	45	–	–	20	65
	Kırklareli	2	–	–	–	–	–	72	71	143
	Tekirdağ	1	–	–	–	–	–	–	44	44
	**Total**	**29**	**27**	**17**	**1**	**93**	**125**	**178**	**149**	**590**
Agean	Afyon	3	1	6	–	–	–	–	–	7
	Aydın	1	–	–	10	–	–	–	–	10
	Denizli	1	–	–	–	–	–	–	20	20
	İzmir	21	–	30	30	55	–	74	–	189
	Kütahya	5	5	–	–	–	–	–	–	5
	**Total**	**31**	**6**	**36**	**40**	**55**	–	**74**	**20**	**231**
Middle Anatolian	Eskişehir	1	–	–	21	–	–	–	–	21
	Kırşehir	1	–	–	–	–	–	43	–	43
	Konya	1	–	–	–	–	36	–	–	36
	**Total**	**3**	–	–	**21**	–	**36**	**43**	–	**100**
Black Sea	Bolu	6	8	–	–	–	–	–	–	8
	Samsun	6	6	–	–	–	–	–	–	6
	**Total**	**12**	**14**	–	–	–	–	–	–	**14**
Southeast	Gaziantep	1	–	–	–	–	–	–	91	91
	**Total**	**1**	–	–	–	–	–	–	**91**	**91**
	**TOTAL**	**76**	**47**	**53**	**62**	**148**	**161**	**295**	**260**	**1026**

*Note*: GI: dairy farms with < 20 cows; GII: dairy farms with 21–100 cows; GIII: dairy farms with 101–200 cows; GIV: dairy farms with 201–300 cows; GV: dairy farms with 301–500 cows; GVI: dairy farms with 501–1000 cows; GVII: dairy farms with > 1000 cows. Bold values represent total figures at both regional and national levels.

Herds were categorised into seven groups according to the number of lactating cows in the herd at the time of sampling; Group I (≤ 20 cows), Group II (21–100 cows), Group III (101–200 cows), Group IV (201–300 cows), Group V (301–500 cows), Group VI (501–1000 cows) and Group VII (> 1000 cows) in Table 1. There were 29, 19, 8, 4, 4, 6, 6 herds in each group, respectively. In total, 47, 53, 62, 148, 161, 295, 260 calves were sampled in each group. The percentage of calves sampled/total calves born/herd decreased as herd size increased: Group I (5%–100%), Group II (2%–48%), Group III (1%–12%), Group IV (6%–18%), Group V (8%–16%), Group VI (3%–14%) and Group VII (0.5%–7%).

Since dairy cattle farms in Türkiye are mostly located in Marmara, Aegean and Central Anatolia regions, most of the samples were collected from these regions (Ministry of Agriculture and Forestry 2025).

### 2.2 Risk Factor Variables

In addition to herd size, parity, colostrum feeding method, amount of colostrum fed, season of birth (spring = March–May; summer = June–August, autumn = September–November, winter = December–February), calf age, sex and identification number of the calves included in the study were obtained from the documents on the farms.

Information on parity, calf breeds, amount of colostrum given, season of birth and calf age are shown in Tables 2, 3, 4, 5, 6, respectively.

### 2.3 Sample Collection and Testing

Blood samples were taken from all 1‐ to 5‐day‐old calves on the farm on the day of sampling. The number of calves sampled/herd usually reflected the herd size. In the event that a calf was visibly sick or dehydrated at the time of sampling, it was excluded from the study. Each enrolled calf was identified by an official ear tag, and 6–8 mL of whole blood was collected from the jugular vein into a sterile 9 mL vacutainer (Vacuette Tube 9 mL CAT serum clot activator anticoagulant‐free blood tube İzmir Türkiye) by the local veterinarian.

Blood samples were centrifuged at 4500 × g for 5 min at 20°C within 2–4 h of collection. Following centrifugation, the serum samples were separated and frozen at −20°C. They were then transported to the Faculty of Veterinary Medicine at Uludağ University under cold‐chain conditions. The resulting serum was examined using a digital Brix refractometer (MilwaukeeMA882 Digital Brix Refractometer, Milwaukee Electronics Kft., Szeged, Hungary), and a Brix reading of < 8.5% was considered to be FPTI (Hernandez et al. 2016). In addition, overall Brix values were categorised as excellent, good, fair and poor (Lombard et al. 2020).

#### 2.2.2. Statistical Analysis

The statistical analyses were performed using IBM SPSS software (IBM Corp. 2015, IBM SPSS Statistics for Windows, version 23.0. IBM Corp, Armonk, NY, USA).

The effects of dam, calf and herd management variables on the occurrence of FPTI were examined through the application of logistic regression analysis. In determining the variables to be included in the model, first, univariate analyses were performed for each risk factor, and the variables to be included in the model were determined by accepting the value of *p* < 0.20 as significant. The Brix variable was selected as the dependent variable with binary values (Brix < 8.5 and Brix ≥ 8.5). The following factors were analysed as potential risk factors (independent variables): breed, whether the dam is a primiparous or a multiparous, colostrum administration method, herd size, calf age, region of farm location and birth time. Risk factors were excluded from multivariable modelling using a backwards stepwise elimination process. The statistical significance level was set at *p* < 0.05 for interpretation.

## 3 Results

### 3.1 Overall

A total of 1026 calf serum samples were evaluated by digital Brix refractometer, and the mean (SE) Brix value was 9.37 ± 0.04. The calf‐level incidence of FPTI was 230/1026 (22.4%). When Brix values were categorised as excellent, good, fair and poor, the distribution of results was: 48.3%, 17.6%, 18.0% and 15.9%, respectively (Table 7).

**TABLE 7:** Different categorised results of Brix values in herd size groups and in all calves.

		Group I		Group II		Group III		Group IV		Group V		Group VI		Group VII		Total	
**Categories**	**Brix %**	*n*	*%*	*n*	*%*	*n*	*%*	*n*	*%*	*n*	*%*	*n*	*%*	*n*	*%*	*n*	*%*
Excellent	≥ 9.4	19	*40.4*	17	*32.1*	32	*51.6*	64	*43.2*	63	*39.2*	159	*53.9*	142	*54.6*	496	*48.3*
Good	8.9–9.3	5	*10.6*	7	*13.2*	4	*6.4*	22	*14.8*	41	*25.4*	35	*11.8*	67	*25.8*	181	*17.6*
Fair	8.1–8.8	11	*23.5*	12	*22.6*	17	*27.5*	37	*25.2*	15	*9.3*	62	*21.1*	31	*11.9*	185	*18.0*
Poor	< 8.1	12	*25.5*	17	*32.1*	9	*14.5*	25	*16.8*	42	*26.1*	39	*13.2*	20	*7.7*	164	*15.9*
	**Total**	47	*100*	53	*100*	62	*100*	148	*100*	161	*100*	295	*100*	260	*100*	1026	*100*

*Note*: GI: dairy farms with < 20 cows; GII: dairy farms with 21–100 cows; GIII: dairy farms with 101–200 cows; GIV: dairy farms with 201–300 cows; GV: dairy farms with 301–500 cows; GVI: dairy farms with 501–1000 cows; GVII: dairy farms with > 1000 cows.

### 3.2 Effects of Risk Factors

### 3.3 Herd Size Group

There was a significant effect of herd size group on occurrence of FPTI (*p* = 0.002) (Table 8). The lowest prevalence of FPTI was in the largest herd size group (Group VII; 12.3%), and the highest prevalence was in the second smallest herd size group (Group II; 43.4%; Table 9). There was also a significant effect of herd size group on Brix value (Table 9). However, both the lowest (8.80 ± 0.18 in Group II) and the highest (9.74 ± 0.23 in Group III) group mean Brix values were in adjacent herd group size categories (21–100 and 101–200 cows), respectively. As can be seen in Table 7, the highest excellent rate (54.6%) was found in Group VII, and the highest poor rate (32.1%) was found in Group II.

**TABLE 8:** Final multilevel logistic regression model of risk factors associated with FPTI (serum Brix value < 8.5%) in 1026 calves on 76 Turkish dairy farms.

Variable	Estimate	SE	OR	Lower bound	Upper bound	*p*‐value
**Intercept (constant)**	1.026	0.508	2.791	–	–	0.043
**Herd size (cows)**						0.002
Group I (≤ 20)	Referent					
Group II (21–100)	−0.979	0.675	0.376	0.100	1.412	0.147
Group III)101–200)	−0.219	0.813	0.803	0.163	3.954	0.787
Group IV (201–300)	0.329	0.753	1.390	0.318	6.087	0.662
Group V (301–500)	−0.410	0.730	0.664	0.159	2.775	0.575
Group VI (501–1000)	0.254	0.676	1.289	0.343	4.847	0.707
Group VII (> 1000)	0.777	0.728	2.175	0.522	9.064	0.286
**Parity**	0.520	0.186	1.682	1.168	2.423	0.005
**Breed of the calves**						< 0.001
Holstein	Referent					
Simmental	−1.456	0.299	0.233	0.130	0.419	< 0.001
Crossbreed	0.258	0.876	1.295	0.233	7.206	0.768
Jersey	−0.757	0.419	0.469	0.206	1.067	0.071
**Colostrum feeding method**						0.003
Feed with a bottle	Referent					
Feed with a catheter	−1.087	0.529	0.337	0.119	0.951	0.040
Feed by suckling their dam	−1.328	0.511	0.265	0.097	0.721	0.009
**Season of birth**						0.005
Spring	Referent					
Summer	−1.126	0.556	0.324	0.109	0.963	0.043
Autumn	−0.081	0.444	0.922	0.386	2.201	0.855
Winter	−0.998	0.422	0.369	0.161	0.843	0.018
**Age of the calves**	−0.209	0.123	0.811	0.638	1.033	0.089
**Region**						0.604
Marmara	Referent					
Agean	0.051	0.331	1.052	0.549	2.014	0.879
Middle Anatolian	−0.096	0.372	0.909	0.439	1.883	0.797
Southeast	0.520	0.545	1.682	0.578	4.892	0.340
Black Sea	1.396	1.084	4.041	0.483	33.794	0.197

Abbreviations: FPTI = failure of passive transfer of immunity; OR = odds ratio; SE = standard error of the estimate.

**TABLE 9:** Effect of herd size group on mean Brix values and on prevalence of FPTI.

	Group I	Group II	Group III	Group IV	Group V	Group VI	Group VII	Total
Number of calf serum samples	47	53	62	148	161	295	260	1026
Mean (SE) Brix %	8.93 ± 0.21	8.80 ± 0.18	9.74 ± 0.23	9.27 ± 0.10	8.97 ± 0.10	9.53 ± 0.08	9.59 ± 0.07	9.37 ± 0.04
Calves with an FPTI (*n*)	16	23	16	31	46	66	32	230
Calves with FPTI (%)	34.04	43.39	25.80	20.94	28.57	22.37	12.30	22.42

*Note*: GI: dairy farms with < 20 cows; GII: dairy farms with 21–100 cows; GIII: dairy farms with 101–200 cows; GIV: dairy farms with 201–300 cows; GV: dairy farms with 301–500 cows; GVI: dairy farms with 501–1000 cows; GVII: dairy farms with > 1000 cows.

### 3.4 Dam Parity Group

There was a significant effect of dam parity group on occurrence of FPTI (OR = 1.682; *p* = 0.005; Table 8). More calves from primiparous (29.6%) had FPTI than calves of multiparous (17.1%; Table 2). The proportion of primiparous and multiparous cattle in the study was 42.5% and 57.50%, respectively.

**TABLE 2:** Classification of dairy cattle in the groups according to their primiparous and multiparous number.

	Group I	Group II	Group III	Group IV	Group V		Group VI	Group VII	Total
Primiparous *n* % FPTI *n* %	12 25.53 3 25	22 41.50 9 40.90	32 51.61 12 37.5	70 47.29 26 37.14	88 54.65 30 34.09	124 42.03 40 35.25		88 33.84 9 10.22	436 42.49 12,9 29.58
Multiparous *n* % FPTI *n* %	35 74.46 13 37.14	31 58.49 14 45.16	30 48.38 4 13.33	78 52.70 5 6.41	73 45.34 16 21.91	171 57.96 26 15.20		172 66.15 23 13.37	590 57.50 101 17.11
Sample *n*	47	53	62	148	161	295		260	1026

*Note*: GI: dairy farms with < 20 cows; GII: dairy farms with 21–100 cows; GIII: dairy farms with 101–200 cows; GIV: dairy farms with 201–300 cows; GV: dairy farms with 301–500 cows; GVI: dairy farms with 501–1000 cows; GVII: dairy farms with > 1000 cows.

**TABLE 3:** Distribution of FPTI rates in calf breeds according to groups.

	Group I	Group II	Group III	Group IV	Group V	Group VI	Group VII	Total
Holstein n	14	41	60	195	50	204	268	832
FPTI *n* %	4 28.57	21 51.21	16 26.66	12 6.15	42 84.00	23 11.27	27 10.07	145 17.42
Simmental *n*	28	7	1	–	3	67	8	114
FPTI *n* %	11 39.28	1 14.28	‐ ‐	‐ ‐	3 100.00	43 64.17	3 37.50	61 53.50
Jersey *n*				58	2		6	66
FPTI *n* %	‐ ‐	‐ ‐	‐ ‐	19 32.75	1 50.00	‐ ‐	2 33.33	22 33.33
Crossbreed n	7	3	1				3	14
FPTI *n* %	1 14.28	1 33.33	‐ ‐	‐ ‐	‐ ‐	‐ ‐	‐ ‐	2 14.28
								1026
Total FPTI	16	23	16	31	46	66	32	230

*Note*: GI: dairy farms with < 20 cows; GII: dairy farms with 21–100 cows; GIII: dairy farms with 101–200 cows; GIV: dairy farms with 201–300 cows; GV: dairy farms with 301–500 cows; GVI: dairy farms with 501–1000 cows; GVII: dairy farms with > 1000 cows.

### 3.5 Calf Breed

There was a significant effect of dam parity group on the occurrence of FPTI (*p* < 0.001) (Table 8). When compared with the Holstein breed, the Simmental breed had a higher prevalence of FPTI (*p* < 0.001; Table 8).

The average Brix value and FPTI rates for Holstein, Simental, Jersey, and Crossbred cattle were 9.55 ± 0.05 and 17.4%, 8.50 ± 0.12 and 53.5%, 8.63 ± 0.11 and 33.3%, and 9.43 ± 0.41 and 14.3%, respectively. It was noteworthy that the highest FPTI rate was in Simmental calves, and these calves were mostly found in Group VI (64.17%).

### 3.6 Colostrum Feeding Method

In the study, 21.21% of the calves in Group I, 19.56% of the calves in Group II and 29.03% of the calves in Group III were fed by suckling their mothers, and only 15.38% of the calves in Group VII were fed with a catheter. All other calves consumed colostrum by bottle feeding.

There was a significant effect of feeding method on the occurrence of FPTI (*p* = 0.04; Table 8). When the calves were compared according to how they received colostrum, the prevalence of FPTI was lower in bottle‐fed calves or calves fed with a stomach tube, compared to those left with their mothers.

### 3.7 Colostrum Volume Fed

The average amount of colostrum consumed by calves on the first day is shown in Table 3.

**TABLE 4:** Average amount of colostrum consumed by calves on the first day according to groups.

The amount of colostrum consumed	Group I	Group II	Group III	Group IV	Group V	Group VI	Group VII
In the first 2 h (L)	2.5 ± 0.27	2.08 ± 0.16	2.5 ± 0	2.50 ± 0	2.65 ± 0.62	2.90 ± 0.29	3.20 ± 0.3
In the first 24 h (L)	5.30 ± 0.27	4.93 ± 0.3	6 ± 1.00	6.33 ± 0.66	6.75 ± 0.75	6.87 ± 1.08	7.75 ± 0.62

*Note*: GI: dairy farms with < 20 cows; GII: dairy farms with 21–100 cows; GIII: dairy farms with 101–200 cows; GIV: dairy farms with 201–300 cows; GV: dairy farms with 301–500 cows; GVI: dairy farms with 501–1000 cows; GVII: dairy farms with > 1000 cows.

**TABLE 5:** Distribution of calves with FPTI according to seasons.

	Group I	Group II	Group III	Group IV	Group V	Group VI	Group VII	Total
Spring *n* % FPTI n %	19 40.42	13 24.52		3 2.02		31 10.5	30 11.53	96 9.35
	6 31.57	3 23.07				4	3 10	16 16.66
Summer *n* % FPTI *n* %		5 9.43	6 9.67	26 17.56			9 3.46	46 4.48
			5 83.33	8 30.76				13 28.26
Autumn *n* % FPTI *n* %	12 25.53	23 43.39	5 8.06	53 35.81	85 52.79	91 30.84	72 27.69	341 33.23
	2 16.66	13 56.52	2 40		19 22.35	7 7.69	5 6.94	48 14.07
Winter *n* % FPTI *n* %	16 34.04	12 22.64	51 82.25	66 44.59	76 47.20	173 58.64	149 57.30	543 52.92
	8 50	7 58.33	9 17.64	23 34.84	27 35.52	55 31.79	24 16.1	153 28.17
Total *n* FPTI *n*	47	53	62	148	161	295	260	1026
	16	23	16	31	46	66	32	230

*Note*: GI: dairy farms with < 20 cows; GII: dairy farms with 21–100 cows; GIII: dairy farms with 101–200 cows; GIV: dairy farms with 201–300 cows; GV: dairy farms with 301–500 cows; GVI: dairy farms with 501–1000 cows; GVII: dairy farms with > 1000 cows.

**TABLE 6:** FPTI distribution according to days.

Day	Day	Group I	Group II	Group III	Group IV	Group V	Group VI	Group VII	Total
1	*n* % FPTI *n* %	6 12.76	16 30.18	6 9.67	5 3.37		21 7.11	7 2.69	61 5.94
		1 16.66	7 43.75		3 60		3 14.28	1 14.28	15 24.59
2	*n* % FPTI *n* %	14 29.78	16 30.18	33 53.22	13,7 92.56	75 46.58	26,9 91.18	19,0 73.07	73,4 71.53
		3 21.42	3 18.75	3 9.09	26 10.97	30	62 23.04	22 11.57	14,9 20.29
3	*n* % FPTI *n* %	16 34.04	11 20.75	16 25.8	1 0.67	86 53.41	2 0.67	24 9.23	156 15.2
		6 37.5	5 45.45	6 37.5	1 100	16		1 4.16	35 22.43
4	*n* % FPTI *n* %	6 12.76	5 9.43	5 8.06	3 2.02		2 0.67	29 11.15	50 4.87
		3 50	4 80	5 100			1 50	4 13.79	17 34
5	*n* % FPTI *n* %	5 10.63	5 9.43	2 3.22	2 1.35		1 0.33	10 3.84	25 2.43
		3 60	4 80	2 100	1 50			4 40	14 56
	Total *n* FPTI *n*	47	53	62	148	161	295	260	1026
		16	23	16	31	46	66	32	230

*Note*: GI: dairy farms with < 20 cows; GII: dairy farms with 21–100 cows; GIII: dairy farms with 101–200 cows; GIV: dairy farms with 201–300 cows; GV: dairy farms with 301–500 cows; GVI: dairy farms with 501–1000 cows; GVII: dairy farms with > 1000 cows.

### 3.8 Season of Birth

The highest number of samples was collected in winter, and the highest rates of calves with FPTI were observed in summer and winter. There was a significant effect of birth season on the occurrence of FPTI (*p* = 0.005; Table 8). Relative to calves born in spring, calves born in summer (*p* = 0.043) and winter (*p* = 0.018) had a higher prevalence of FPTI (Table 8).

### 3.9 Calf Sex

There was no significant effect of calf sex on the occurrence of FPTI. The mean (SE) Brix % value of male calves (*n* = 228) was 9.02 ± 0.09, and for female calves (*n* = 466) was 9.21 ± 0.06. Data on calf sex were not recorded in all cases.

### 3.10 Calf Age at Sampling

There was no significant effect of calf age at sampling on the occurrence of FPTI (Table 8). The majority of samples were collected on the second and third days (890 calves). When the rates of calves with FPTI were distributed according to days, it was observed that the rate of FPTI appeared to increase on Days 4 and 5, but the number of calves sampled on these days (75) was much smaller than on Days 1–3 (951).

### 3.11 Farm Region

There was no significant effect of farm regional location on the occurrence of FPTI (Table 8).

## 4 Discussion

### 4.1 Overall

The percentage of calves with FPTI was 22.4%. The prevalence of FPTI has been documented as 38% of 1018 calves from 100 herds in Australia (Vogels et al. 2013) and in 34% of 882 calves from 49 herds in the United States (Wenz 2011). Staněk et al. (2019) found FPTI rate 34.06% in a study of 1175 calves in 33 dairy farms. Lombard et al. (2020) reported that an FPTI prevalence of less than 10% is acceptable at the herd level on dairy farms. In our study, the rate of poor Brix value was found to be 15.9% higher than the FPTI rate of Lombard et al. (2020). As the size of the herd increases, the poor Brix values decrease. In fact, in Group VII (*n* > 1000), the poor Brix values fall below 10% even as the herd size increases. Haggerty et al. (2021) found FPTI rate 14.05% in a study of 370 calves in 38 dairy farms. Thus, the relatively high prevalence of FPTI in the present study is a concern given this international benchmark. In Türkiye, previous studies on FPTI were conducted in fewer regions and with a smaller number of calves. Previous studies have demonstrated considerable variation in FPTI rates, with reported values of 29.3%, Topal and Batmaz (2020), 50% Demir and Tütüncü (2022), 17.25% (Kara and Ceylan (2021) and 10.4% Akköse et al. (2022a). The high variability in the results of previous studies on the prevalence of FPTI in Türkiye may be due to the fact that it was measured in a small number of calves locally.

The overall average BRIX value in the present study was 9.37. Similar average values have been reported in international studies: 9.2% by Hernandez et al. (2016); 8.2% by Sutter et al. (2020); 8.75% by Elsohaby et al. (2019). In a study conducted by Akköse et al. (2022a) on 260 dairy calves in Türkiye, the average Brix value was found to be 9.11%; Topal et al. (2018) found an average Brix value of 9.61% in 60 calves. The average Brix value in the present study was similar to those in previous studies in Türkiye.

### 4.2 Herd Size

Group I (< 20 cows; 34.04%) and Group II (21–100 cows; 43.39%) were the two groups with the highest proportion of calves with FPTI; this is consistent with the fact that the average Brix values of the calves in the two groups were the lowest. This suggests that calves in smaller herds have a greater risk of FPTI. This was not associated with a relatively higher prevalence of primiparous cows in these herds.

It can be concluded that colostrum management of calves on farms with 21–100 cows is not done sufficiently or should be done more carefully. In the study conducted by Kehoe et al. (2007), it was reported that there were differences in factors such as dry period protocols, measurement of colostrum quality, use of pasteurisation, its storage, amount and time of colostrum delivery in small farms (under 100 cows), while in large farms (above 200 cows), factors such as management protocols, high number of workers and monitoring of its intake in the first hours of the calf born were better followed. In a study reported by Staněk et al. (2019), farms were classified into four groups based on their cow population: < 200, 200–399, 400–599 and ≥ 600 cows. The findings indicated that the IgG content in calf sera exhibited a positive correlation with herd size. It was highlighted that the presence of dedicated farm personnel solely responsible for the care of newborn calves is a crucial element influencing FPTI (Staněk et al. 2019). The findings of these studies, which indicate that the FPTI rate is lower in larger enterprises, are in alignment with the results of our own study.

Group VI had the highest number of Simmental calves, which may have caused the proportion of calves with FPTI (22.37%) in Group VI to increase. Since the birth weights of Simmental calves were higher than the birth weights of Holstein calves, the amount of colostrum given may not have been sufficient (Batmaz 2021).

Average Brix values between the groups are compared, and in Group III, Group VI and Group VII, the average Brix values of calf sera were above 9.5%. This rate was less than 9% in Group I, Group II and Group V. Group I and Group II, which had the lowest number of animals, had the lowest average Brix values. This may be attributed to the fact that approximately 20% of the calves within these groups were able to obtain colostrum through suckling their mothers.

### 4.3 Dam Parity

When mothers of calves included in the study were classified as primiparous or multiparous, the FPTI rate in calves from primiparous cows (29.58%) was found to be higher than in multiparous cows (17.11%). As in some previous studies, it is an expected result that colostrum quality is lower in primiparous cows compared to multiparous cows (Weaver et al. 2000; Dunn et al. 2017).

When the mean serum Brix values were evaluated according to dam parity, it was found that the values were higher in multiparous cows. Previous studies have also reported that the IgG, total protein and Brix averages of multiparous cows are higher than those of primiparous cows (Avendaño‐Reyes et al. 2024; Meyer and Rathert‐Williams2024). This difference may be attributed to the higher colostrum quality observed in multiparous cows (Godden et al. 2019; Lima et al. 2024).

### 4.4 Breed

When the FPTI rates of the calves in the study were evaluated according to their breed, it was found that the highest rate (53.50%) was observed in Simmental calves. This breed was followed by Jersey (33.33%) and Holstein (17.42%) calves. Previous studies have reported that the quality of colostrum may differ according to cow breed (Godden et al. 2019; Kessler et al. 2020). In a study conducted by Cavirani et al. (2024), the influence of breed on colostrum and passive transfer was investigated in five distinct breeds: Italian Friesian, Reggiana and Bianca Modenese, Piemontese and Limousine. The findings indicated that breed plays a role in colostrum and the passive transfer immunity process (Cavirani et al. 2024). Villarroel et al. (2013) compared the passive transfer status of Jersey and Holstein calves and showed that serum IgG levels in Jersey calves were higher than Holsteins and concluded that different cattle breeds need their own established reference values. In our study, the high rate of FPTI in Jersey calves in the two farms where samples were collected may be explained by inadequacies in management practices in those farms.

Akköse et al. (2022b) reported the Brix threshold value as 7.9% against 10 g/L IgG level in calf serum in their study conducted in Simmental breed calves. Although the Brix threshold value was higher in our study (8.5%), the high rate of FPTI in Simmental calves can be accepted as an indicator that it may be related not only to breed but also to other factors (birth weight, season, colostrum volume and quality).

### 4.5 Volume of Colostrum Fed

The amount of colostrum consumed by calves in the first 24 h after birth is very important to prevent FPTI. It has been suggested that it should be milked as soon as possible after birth (Morin et al. 2010) and administered to the calf immediately, with an average volume of 1–4 L. When the colostrum consumption of the calves in the first 24 h in the present study was compared between the groups, it was seen that Group VII had the highest colostrum consumption. Although colostrum consumption in the first 2 h in Group I and Group II was comparable to that observed in the other groups, the mean intake over the 24‐h period was lower than that of the other groups. This indicates that colostrum consumption in the first 2 h is important, but the total amount of it consumed in the first 24 h is also very important for FPTI (Table 3). It was observed that both colostrum intake and Brix levels increased with farm size. Similar studies have also reported a relationship between the low amount of it received and the risk of FPTI (Phipps et al. 2018; Fernandez‐Novo et al. 2025). Based on these findings, it can be concluded that the amount of colostrum received may be associated with the risk of FPTI, emphasising the importance of early and adequate colostrum intake for passive immunity transfer (Phipps et al. 2018; Fernandez‐Novo et al. 2025).

### 4.6 Colostrum Feeding Method

The average Brix values of calves fed with a bottle were 9.40 ± 0.04, while those fed with an oesophageal tube were 9.35 ± 0.18, and those that suckled from their mothers had an average of 8.25 ± 0.25. Factors affecting colostrum (colostrum quality, fresh colostrum, frozen or pasteurised colostrum) are important because they indirectly affect FPTI, but these data could not be described in detail in the study because detailed information could not be collected from the farms. When evaluating the feeding methods, the amount of colostrum received was known in calves fed with a bottle and oesophageal tube, whereas the amount of colostrum received by suckling calves is unclear (Godden et al. 2019). In suckling calves, the FPTI rate was found to be higher, compared to other feeding methods, possibly due to the unknown quantity and quality of colostrum received. While oesophageal tube feeding may seem advantageous, it should be noted that it is a difficult procedure and carries risks such as aspiration, which could lead to complications (Phipps et al. 2018). However, some studies found no difference (Laestander 2016).

### 4.7 Season

When the collected calf serum Brix values were classified according to the seasons, the FPTI rate was high in summer (28.26%) and winter (28.17%), and therefore the percentage of FPTI was higher in summer and winter than in spring and autumn. In a study conducted by Gulliksen et al. (2008), it was reported that cows calving during the winter months exhibited reduced colostral IgG levels. In another study, Tao et al. (2012) observed that exposure of pregnant cows to heat stress during the dry period had an adverse effect on fetal development and calf immune function. A study by Laporta et al. (2017) reported that pregnant cows exposed to heat stress and calves born to them had lower birth weights, spent less time standing in the first week and had lower serum IGF‐1 levels than cows and calves born to cows with cooling systems, despite having the same suckling reflex. It was reported that exposure to heat stress at the end of gestation negatively affected passive transfer and growth (Laporta et al. 2017). If the number of calves born in summer were as high as in winter, our FPTI rates would be even higher.

When evaluating by season, Çolakoğlu et al. (2021) reported that the IgG concentration in colostrum was lower in the summer, compared to winter, and Avendaño‐Reyes et al. (2024) found that high temperature–humidity index adversely affects milk yield. Additionally, calves born in winter from multiparous cows are more likely to experience FPTI (Moreira et al. 2024).

### 4.8 Age of Calf

The proportion of calves exhibiting FPTI was 20.29% and 22.43% at 48 and 72 h, respectively, when the calf sera were classified according to days. The highest prevalence of calves with FPTI was observed on Days 4 and 5. This finding is consistent with the results of previous studies that have demonstrated that immunoglobulins can be absorbed up to 24 h after birth and that there is an increase in IgG breakdown over the following days (Weaver et al. 2000; Godden 2008; Correa et al. 2022). This result supports the hypothesis that the maximum colostral IgG concentration in calf sera is reached 48 to 72 h after parturition (Lombard et al. 2020).

Although daily age was found to have no effect on FPTI in this study, it has been shown in our previous study that Brix value may have different cut‐off values according to daily age (Topal et al. 2018). Since IgG absorption occurs in the intestines until the 24th hour after birth, it may be more appropriate to measure the Brix value after the 24th–48th hour for PTI status (Godden 2008). Although not statistically significant, this difference may be due to the fact that the amount of IgG starts to decrease from Day 3 (Lopez et al. 2020; Goetz et al. 2025).

### 4.9 Sex

Previous studies have indicated that female calves may be more susceptible to FPTI than males (Lora et al. 2018) or that sex may not be a contributing factor in the development of FPTI (Renaud et al. 2020; Aleri et al. 2021). There was no significant effect of calf on the occurrence of FPTI (Table 8).

The results of the study demonstrated that the mean Brix values were higher in female calves than in male calves, but no statistical difference was found. The findings of this study align with those of some previous research in this field (Vogels et al. 2013; Bragg et al. 2020). This may be due to the fact that the birth weight of male calves is higher than that of females, and they consume the same amount of colostrum. In addition, the fact that 40.35% of the male calves were collected from Groups VI and VII may also have had an effect.

### 4.10 Region

The distribution of FPTI and the Brix averages by region was as follows: Marmara, 25.4% and 9.17 ± 0.05; Aegean, 20.77% and 9.58 ± 0.09; Central Anatolia, 24% and 9.20 ± 0.13; Southeastern Anatolia, 5.49% and 10.13 ± 0.11; and the Black Sea, 21.42% and 9.82 ± 0.43. Since the Eastern Anatolia region has mostly small, pasture‐based family enterprises, no examples could be taken. Negative responses were received from two dairy farms in the Mediterranean region. The aim of the study was to determine the FPTI rate in dairy farms of different sizes. Therefore, we believe that sampling 76 farms in five different regions is representative of the FPTI rate in Türkiye. There is no study of this magnitude in Türkiye so far. In similar studies conducted in Türkiye, the FPTI percentage in Marmara was found to be 30% and 10.4% (Abdullahoğlu et al. 2019; Topal and Batmaz 2020), in the Aegean 17.25% (Akköse et al. 2022a), in Central Anatolia 20% (Kara and Ceylan 2021) and in the Black Sea 50% (Demir and Tütüncü 2022). The fact that an equal number of samples could not be collected from all regions may have caused no difference on the basis of the province and region where the enterprises are located. In addition, it may be necessary to collect more data (especially climate, altitude, etc.) about the regions where the samples were collected. These issues should be investigated in more detail in future studies.

### 4.11 Multiple Factors

In our study, multiple factors such as the size of the farm, the method of colostrum administration, birth of the season and the age of the mother were found to affect the prevalence of FPTI in calves. However, caution should be taken when generalising about FPTI based on these factors alone, for example, the quality of colostrum (the amount of IgG it contains) is as important as the method of administration (Godden et al. 2019; Lombard et al. 2020; Lora et al. 2018). Although the season seems to be effective, the good environmental conditions (such as ambient temperature and humidity) in which the calves are kept may override this factor (Tao et al. 2012; Laporta et al. 2017). Among these factors, the one that can be considered the most general and accurate is that colostrum from multiparous cows contains a higher concentration of IgG (Godden et al. 2019; Lichtmannsperger et al. 2023).

## 5 Conclusion

The prevalence of FPTI in this study was similar to reports internationally. This research shows that there were both modifiable and non‐modifiable significant risk factors for FPTI in Türkiye dairy herds, specifically, colostrum feeding method and season of birth and herd size, dam parity and calf breed. In future studies, detailed evaluation can be made at the scale of large farms (*n* > 300), and the findings from this study can serve as a source for future research.

## Author Contributions

**Nezaket Sağlam**: data curation, investigation, resources, visualisation, writing – original draft, writing – review and editing. **Onur Topal**: data curation, investigation, methodology, resources, visualisation, writing – original draft, writing – review and editing. **Piraye Biçer**: investigation, resources. **Ender Uzabaci**: formal analysis, validation, visualisation. **Hıdır Gençoğlu**: investigation, resources, visualisation. **John F. Mee**: methodology, supervision, visualisation, writing – original draft, writing – review and editing. **Hasan Batmaz**: data curation, investigation, methodology, project administration, supervision, visualisation, writing – original draft, writing – review and editing.

## Funding

This research is supported by Bursa Uludag Universitesi.

## Ethics Statement

This study was approved by the Local Ethical Committee for Animal Research of Bursa Uludag University (2023‐ 13 /04).

## Conflicts of Interest

The authors declare no conflicts of interest.

## Data Availability

The data that support the findings of this study are available from the corresponding author upon reasonable request.
